# Mapping and Validation of Major Quantitative Trait Loci for Resistance to Northern Corn Leaf Blight Along With the Determination of the Relationship Between Resistances to Multiple Foliar Pathogens of Maize (*Zea mays* L.)

**DOI:** 10.3389/fgene.2020.548407

**Published:** 2021-01-29

**Authors:** Hosahally Muddrangappa Ranganatha, Hirenallur Chandappa Lohithaswa, Anand Pandravada

**Affiliations:** ^1^Department of Genetics and Plant Breeding, College of Agriculture – Mandya, University of Agricultural Sciences, Bengaluru, Bengaluru, India; ^2^Corteva Agriscience Pvt. Ltd., Kallinayakanahalli, India

**Keywords:** northern corn leaf blight, sorghum downy mildew, southern corn rust, linkage map, QTL, marker assisted selection

## Abstract

Among various foliar diseases affecting maize yields worldwide, northern corn leaf blight (NCLB) is economically important. The genetics of resistance was worked out to be quantitative in nature thereby suggesting the need for the detection of quantitative trait loci (QTL) to initiate effective marker-aided breeding strategies. From the cross CML153 (susceptible) × SKV50 (resistant), 344 F_2__:__3_ progenies were derived and screened for their reaction to NCLB during the rainy season of 2013 and 2014. The identification of QTL affecting resistance to NCLB was carried out using the genetic linkage map constructed with 194 polymorphic SNPs and the disease data recorded on F_2__:__3_ progeny families. Three QTL for NCLB resistance were detected on chromosomes 2, 5, and 8 with the QTL *qNCLB-8-2* explaining the highest phenotypic variation of 16.34% followed by *qNCLB-5* with 10.24%. QTL for resistance to sorghum downy mildew (SDM) and southern corn rust (SCR) were also identified from one season phenotypic data, and the co-location of QTL for resistance to three foliar diseases was investigated. QTL present in chromosome bins 8.03, 5.03, 5.04, and 3.04 for resistance to NCLB, SDM, and SCR were co-localized, indicating their usefulness for the pyramiding of quantitative resistance to multiple foliar pathogens. Marker-assisted selection was practiced in the crosses CM212 × SKV50, HKI162 × SKV50, and CML153 × SKV50 employing markers linked to major QTL on chromosomes 8, 2, and 10 for NCLB, SDM, and SCR resistance, respectively. The populations were advanced to F_6_ stage to derive multiple disease-resistant inbred lines. Out of the 125 lines developed, 77 lines were tested for their combining ability and 39 inbred lines exhibited high general combining ability with an acceptable level of resistance to major diseases.

## Introduction

Maize (*Zea mays* L.) is a widely cultivated food crop worldwide along with rice and wheat. It also serves as a livestock feed and industrial raw material (Troyer, 2006). Maize was diversified first in the highlands of Mexico where it was domesticated from the wild progenitor teosinte, *Z. mays* spp. *parviglumis* (Matsuoka et al., 2002). Globally, maize (*Z. mays* L.) is cultivated in a wide variety of environments with major cultivation in the warmer parts of temperate regions and in humid–subtropical climate (Dowswell et al., 1996).

Among various biotic constraints, foliar diseases are very important yield-limiting factors worldwide and the prevalence of these diseases varies depending on the region or season (Smith, 1999). About 61 diseases have been recorded on maize in India causing yield losses (Payak et al., 1973; Payak and Sharma, 1985). Northern corn leaf blight incited by *Exserohilum turcicum* (Pass) Leonard and Suggs (Teliomorph = *Setosphaeria turcica* (Luttrell), sorghum downy mildew caused by *Peronosclerospora sorghi* (Weston and Uppal), and southern corn rust caused by *Puccinia polysora* (Underwood) are considered as the most persistent and destructive diseases of field maize (Pratt and Gordon, 2006).

Northern corn leaf blight is prevalent throughout the world and is known to cause more than 50% yield losses (Raymundo and Hooker, 1981; Perkins and Pederson, 1987). Disease is known to appear in regions whenever moderate temperatures and high humidity prevail (Carson, 1999; Smith, 1999). Northern corn leaf blight disease was first reported in India by Butler (1907), and it causes 16–98% reduction in grain and fodder yield (Kachapur and Hegde, 1988; Harlapur et al., 2000).

Among several management options available, cultivation of resistant cultivars is the most practical and cost-effective approach in the management of diseases (Fehr, 1987; Ward et al., 1997). To breed a genotype with a high level of resistance, the inheritance pattern of resistant reaction in the material being handled is a prerequisite. Earlier studies on the genetics of resistance to northern corn leaf blight (Jenkins et al., 1952; Hughes and Hooker, 1971; Hettiarachchi et al., 2009; Chaudhary and Mani, 2010; Ranganatha et al., 2017) suggest that resistance is complex and polygenic in nature. Selection for resistance to foliar diseases is effectively practiced through conventional breeding, where susceptible genotypes under disease pressure can be eliminated before harvest (Ali and Yan, 2012). However, conventional breeding is time-consuming and less feasible due to the complex nature of resistance reaction. This favored the development of molecular tools to assist conventional breeding efforts to breed resistant cultivars. Identification of quantitative trait loci (QTL) is one such tool to help in marker-assisted selection (MAS) of resistant genotypes. In maize, a large amount of valuable information exists with reference to QTL conditioning resistance to foliar diseases like northern corn leaf blight (Welz et al., 1999; Welz and Geiger, 2000; Brown et al., 2001; Ping et al., 2007; Asea et al., 2009; Balint-Kurti et al., 2010; Chung et al., 2010, 2011; Zwonitzer et al., 2010; Poland et al., 2011; Schaefer and Bernardo, 2013; Chen et al., 2016; Shridhar Hegde et al., 2018; Xia et al., 2020). Resistance to multiple diseases conditioned by the same locus is an important consideration in breeding durable resistant genotypes. The information regarding studies on the application of markers linked to QTL for pyramiding quantitative resistance to multiple foliar pathogens in maize is less. Hence, an attempt was made to identify QTL for resistance to northern corn leaf blight (NCLB), to investigate the co-location of QTL for resistance to NCLB with those for sorghum downy mildew (SDM) and southern corn rust (SCR) diseases detected using the same mapping population, and also to develop multiple disease-resistant inbred lines pyramided with major QTL for these three foliar diseases.

## Methodology

### Development of Mapping Population

Based on previous experimental data, two inbred lines, viz. CML153 (susceptible inbred, P_1_) and SKV50 (resistant inbred, P_2_), with contrasting disease reaction against northern corn leaf blight were selected for the development of the mapping population. The selected inbreds were crossed during the rainy season of 2012. The F_1_ generation (CML153 × SKV50) was grown during winter 2012–2013 and self-pollinated. The resulting F_2_ individuals were planted during summer 2013 and selfed to derive 344 F_2__:__3_ families. In each F_2__:__3_ family, the leaves from five randomly selected plants were collected for genotypic analysis. Later, during the rainy season of 2013 and 2014, F_2__:__3_ families were screened for disease reaction against northern corn leaf blight in the national disease nursery maintained at ZARS, V.C. Farm, Mandya.

### Genotyping of F_2__:__3_ Mapping Population

A set of 768 single nucleotide polymorphisms (SNPs) covering whole maize genome^1^ was used for the genotyping of parents, and 199 polymorphic SNPs were identified. The 344 F_2__:__3_ progenies were genotyped with these polymorphic markers. Leaf samples were pooled from five random plants of each F_2__:__3_ family and parents and lyophilized in 96-well plates. Samples were loaded to the Illumina BeadXpress Vera Code Reader for genotyping, according to Illumina protocols^2^. The polymorphism detected by SNP marker was scored as A = homozygous maternal genotype (CML153), B = homozygous paternal genotype (SKV50), H = heterozygote genotype, and - = missing samples.

### Phenotyping of F_2__:__3_ Mapping Population

The 344 F_2__:__3_ families along with the two parental lines were screened against *E. turcicum*, causing northern corn leaf blight of maize. The disease screening was conducted in two seasons during the rainy season of 2013 and 2014 in the national disease screening nursery maintained at Mandya. The experimental area was divided into blocks of 3 m width and 32 m length each accommodating 42 progenies. The F_2__:__3_ progenies were planted in a single row of 3 m length employing the randomized complete block design with two replications. The spacing of 75 cm between rows and 20 cm between plants was provided.

### Screening for Northern Corn Leaf Blight (*E. turcicum*)

The susceptible inbred CM202 was planted as the first row and the last row in each block and also after every 10th progeny. The artificial inoculation procedure developed by Shekhar and Kumar (2012) was employed to ensure uniform disease development. The initial inoculum for artificial inoculation of *E. turcicum* was grown in artificial medium under laboratory conditions. The infected leaf tissues were collected from the diseased plants under natural field conditions, washed thrice with sterile water, cultured on potato dextrose agar medium, and then multiplied on sorghum seeds. For this, the sorghum seeds were soaked overnight and transferred to sterilized conical flasks the next day, and the pathogen inoculum was added. The flasks were shaken once in 2 days, and an equal amount of fresh sorghum seeds was mixed after 1 week. The sorghum seeds with pathogen inoculum were ground to a fine powder, and 1–1.5 g of the ground inoculum was added to the leaf whorl of the test entries, followed by a light spray of water to create humidity and initiate infection. Artificial inoculation was made 20 days after sowing between 3:00 and 6:00 p.m., and inoculation was repeated twice after a 1-week interval. The northern corn leaf blight severity was recorded at the flowering stage, i.e., 60th day after sowing by visualizing the leaf area using a standard scale consisting of five broad categories with intermediate ratings (Payak and Sharma, 1983). Based on the disease score, progenies were classified as resistant (<2.5), moderately resistant (2.5 to <3.0), moderately susceptible (3.0 to <3.5), susceptible (3.5 to <4.0), and highly susceptible (4.0–5.0). The disease score data were converted into percent disease severity by using the formula given by Wheeler (1969).

### Statistical Analysis

#### Phenotypic Data Analysis

##### Transformation of field data

The disease data recorded as percent disease index for northern leaf blight infection ranged from 0 to 100. To make the means and variances independent and normally distributed, the percentage data were subjected to arcsine transformation (Little and Hills, 1978). The analysis of variance was performed on transformed phenotypic data using PROC GLM procedure of SAS package version 9.3. Before pooling the data, Bartlett’s test was conducted to test for homogeneity between environments (Gomez and Gomez, 1984). The components of variance in both the seasons were estimated considering various effects (seasons, replicates, and F_3_ families) as random in the statistical model. As described by Bohn et al. (1996), transformed entry means were used for the combined analysis of variance. The variance components were estimated as per Searle (1971). The heritability (*h*^2^) was calculated following Hallauer and Miranda (1981).

$$h^{2}=\frac{\sigma^{2}\,g}{\frac{\sigma^{2}\,e+\sigma^{2}\,g\,e}{\text{re}}+\frac{\sigma^{2}\,g}{\text{e}}}$$

Where *r* = the number of replications and *e* = the number of environments.

To understand the nature of distribution of F_2__:__3_ progenies with respect to disease incidence, skewness and kurtosis were estimated (Snedecor and Cochran, 1994).

### Linkage Map Construction Using iMAS (GMendel)

We used 199 SNP marker data on 344 F_2__:__3_ progenies for linkage map construction. Five markers showed segregation distortion (SD) and the remaining markers showed expected Mendelian segregation ratio of 1:2:1 as revealed by the χ^2^ test. The linkage analysis was performed with 194 markers using GMendel program of iMAS software. For determining linkage groups, a minimum logarithm of odds (LOD) of 3.0 and maximum recombination fraction of 0.40 were set as threshold values. The unique feature of GMendel 2.0 is that it performs multipoint linkage analysis on populations with complex genetic structures. It generates two point maximum likelihood estimates for all pairwise markers. Based on probability rules, linkage phases are correctly assigned and gene order is estimated using an advance multipoint mapping algorithm. Using a powerful method called the simulated annealing algorithm (SAA), multipoint gene order was determined by GMendel 2.0. The validation of marker ordering was carried out by Monte Carlo and bootstrap methods. Using the Haldane mapping function, recombination fraction was converted into map distance in centimorgan (cM) and the linkage map was constructed using intermarker distances calculated from the GMendel program.

### QTL Location by WinQTL Cartographer Version 2.5

The analysis of QTL controlling the northern corn leaf blight resistance was performed on the arcsine-transformed means of F_2__:__3_ families within each season as well as over seasons. The phenotypic data (rainy season of 2013 and 2014) and genotypic data of 194 SNP markers across 10 chromosomes were used to identify the QTL associated with disease resistance employing WinQTL Cartographer version 2.5 (Wang et al., 2010). The replicated mean data of 344 F_3_ progenies for northern leaf blight were used for QTL mapping in each season. To determine the QTL across the seasons, replicated means of across-season means of 344 F_2__:__3_ progenies were used. The composite interval mapping method (CIM) was used for QTL analysis (Zeng, 1994) employing WinQTL Cartographer 2.5. The presence of putative QTL in an interval was tested using the Bonferroni χ^2^ approximation (Zeng, 1994) corresponding to genome-wise type-I error by using a critical value for the LOD threshold of 2.5. Both additive and dominance models were used for the analysis in this study as the mapping population is comprised of F_2__:__3_ progenies.

The ratio of the absolute value of dominance effect to the absolute value of additive effect, i.e., | *d*| /| *a*|, was used to determine gene action with 0–0.20 = additive, 0.21–0.80 = partial dominance, 0.81–1.20 = dominance, and >1.20 = overdominance (Stuber et al., 1987).

### Co-localization of QTL for Multiple Foliar Diseases of Maize

The QTL for resistance to SDM and SCR were also identified along with NCLB. These QTL were co-localized to different bins of the chromosomes (Gardiner et al., 1993)^3^. Pearson correlation coefficients between the means of northern leaf blight, sorghum downy mildew, and southern corn rust were calculated to assess the degree of association between the foliar diseases of maize.

**Table:** 

*rp* (*xy*)	=	*Covp* (*xy*)/√σ^2^*p* (*x*). σ^2^*p* (*y*
		

Where,

*rp* (*xy*) = Correlation between “*x*” and “*y*”

*Covp* (*xy*) = Covariance between “*x*” and “*y*”

σ^2^*p* (*x*) = Variance of “*x*”

σ^2^*p* (*y*) = Variance of “*y*”

### Development of Inbred Lines With Multiple Disease Resistance and High General Combining Ability

We attempted to develop inbred lines with resistance to three foliar diseases, namely sorghum downy mildew, northern corn leaf blight, and Polysora rust, using the high combining susceptible inbred lines CML153, CM212, and HKI162. These inbreds were crossed to the resistant inbred SKV50 during the 2014 summer season to derive inbred lines. The three F_1_s (CM212 × SKV50, HKI162 × SKV50, and CML153 × SKV50) were raised during the rainy season of 2015 and selfed. During the summer season of 2016, F_2_ plants were raised and screened for three major QTL present on chromosomes 8 (MZA6428-11 and MZA3856-10) for NCLB, 2 (MZA3668-12 and C00324-01) for SDM, and 10 (MZA15331-16 and MZA3922-32) for SCR resistance. The resistant progenies were advanced to the F_6_ stage employing the plant-to-row approach. These progenies were screened in disease nurseries and 125 progenies with acceptable level of disease reaction were identified from three crosses. These progenies were crossed to the open pollinated tester CM500, 77 test cross progenies were obtained and evaluated during the summer season of 2017 in a single row plot of 4 m length with two replications to identify progenies with high general combining ability. The 77 progenies were evaluated for disease reaction during the rainy season of 2018.

## Results

### Phenotypic Data Analysis

Weather conditions at Mandya favored the development of severe northern corn leaf blight disease. The percent disease severity and percent disease index values during the rainy season of 2013 and 2014 ranged from 0 to 100% which followed a binomial distribution. An attempt was made to make the means and variances independent and normally distributed by subjecting the data for arcsine transformation. The arcsine-transformed percent disease data of 344 F_2__:__3_ progenies were used for statistical analysis and QTL mapping.

The parents CML153 and SKV50 differed significantly in their reaction to the NCLB disease as indicated by their percent disease incidence (Table 1). The parent SKV50 showed resistance reaction and CML153 was susceptible. In F_3_ progenies, the mean northern corn leaf blight disease incidence was 52.38% in 2013, 40.71% in 2014, and 46.55% when pooled over seasons. The maximum range of disease incidence (17.14–84.29%) was recorded in 2013 followed by 2014 (16.00–74.00%).

**TABLE 1:** Mean disease incidence, skewness, kurtosis, variance components, and heritability (H) for reaction of F_2__:__3_ progenies to northern corn leaf blight (NCLB) during the rainy season of 2013 and 2014.

**Disease**	**Season**	**Susceptible parent mean**	**Resistant parent mean**	**F_2__:__3_ grand mean**	**Skewness**	**Kurtosis**	**KS test (D)**	**Pr > D**	**Range in F_2__:__3_ progenies**	**Variance components**		**Heritability (%)**
										**δ^2^_*g*_**	**δ^2^_*ge*_**	
Northern corn leaf blight	Rainy season, 2013	66.00	5.00	52.38	0.095	−0.397	0.08	<0.01	17.14–84.29	42.56**		58.82
	Rainy season, 2014	75.00	7.00	40.71	0.188	−0.101	0.063	<0.01	16.00–74.00	24.54**		43.67
	Combined	70.50	6.00	46.55	0.125	−0.362	0.072	<0.01	26.50–69.25		23.57**	65.72

*Significant at *p* = 0.05; **significant at *p* = 0.01.

The analysis of variance revealed significant differences among the progenies indicating the presence of genetic variability in the F_3_ progenies (Table 1). Non-significant Bartlett’s χ^2^ test indicated the homogeneity of error mean sum of squares between seasons, and hence, data were pooled. Variance due to genotype and genotype × season interaction was significant, indicating that the expression of disease incidence significantly varied among F_3_ families and depends upon testing season.

### Genetic Variability Studies in the F_2__:__3_ Population of Maize

The estimates of phenotypic coefficient of variation (PCV) and genotypic coefficient of variation (GCV) for northern corn leaf blight were moderate in 2013 and 2014 (Table 1). High heritability and moderate GAM were noticed in 2013 (58.82 and 19.68%, respectively) and 2014 (43.67 and 16.57%, respectively), whereas in the pooled data, the heritability value was 65.72% and GAM was 17.42%.

### Test for Normality, Skewness, and Kurtosis

**FIGURE 1:**
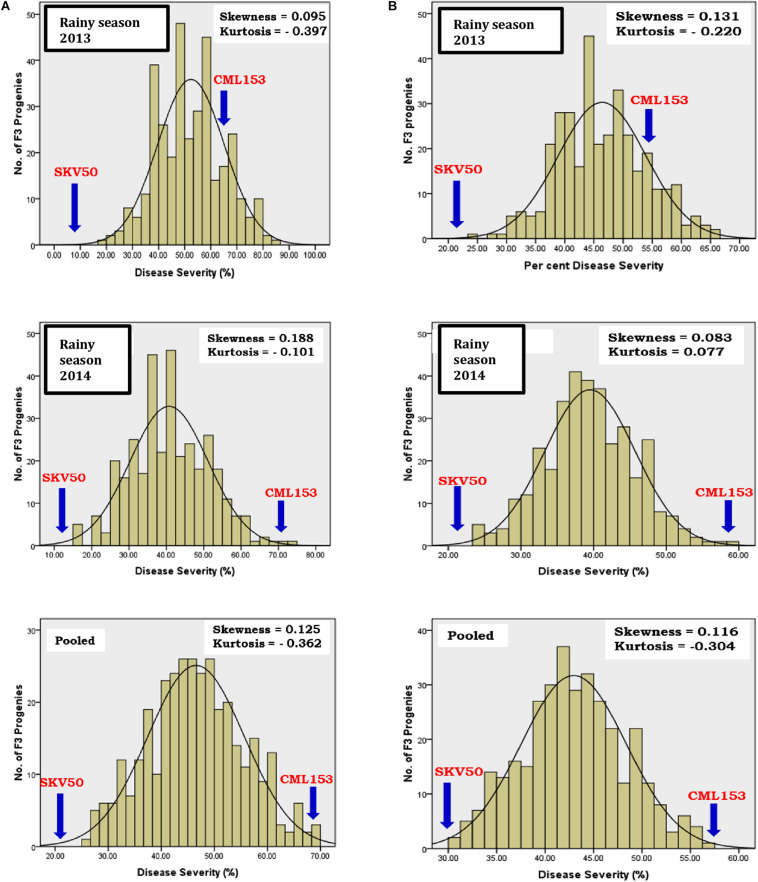
Frequency distribution of mean per cent disease severity of northern corn leaf blight in the F2:3 population derived from the cross CML153 × SKV50 (**A**, original; **B**, Arcsine transformed).

The frequency distribution pattern of F_3_ families was positively skewed and platykurtic for NCLB disease (Figure 1). The results of the test for normality by the Kolmogorov–Smirnov goodness-of-fit test indicated that the distribution of phenotypic means, within and across the seasons, deviated significantly from normal distribution with majority of the progenies skewed toward resistance in northern corn leaf blight.

### Construction of the Linkage Map

Out of the 768 SNP markers, 194 SNP markers were found polymorphic and segregating in Mendelian fashion (1:2:1) and, hence, used for linkage map construction. The markers were mapped on 10 linkage groups (LGs) spanning 2,143.02 cM. The number of markers mapped per linkage group ranged from 6 (LG7) to 33 (LG1). The length of the linkage groups ranged from 152.25 (LG9) to 308.76 cM (LG1) with an average interval distance of 10.77 cM, indicating comparatively high-density SNP linkage map. The SNP map constructed was compared to the maize genome database^4^ ^,5^ and genomically analyzed according to a previous study reported by Jones et al. (2009). This linkage map was used for the identification and mapping of QTL conferring resistance to three foliar diseases of maize.

### QTL Analysis

**FIGURE 2:**
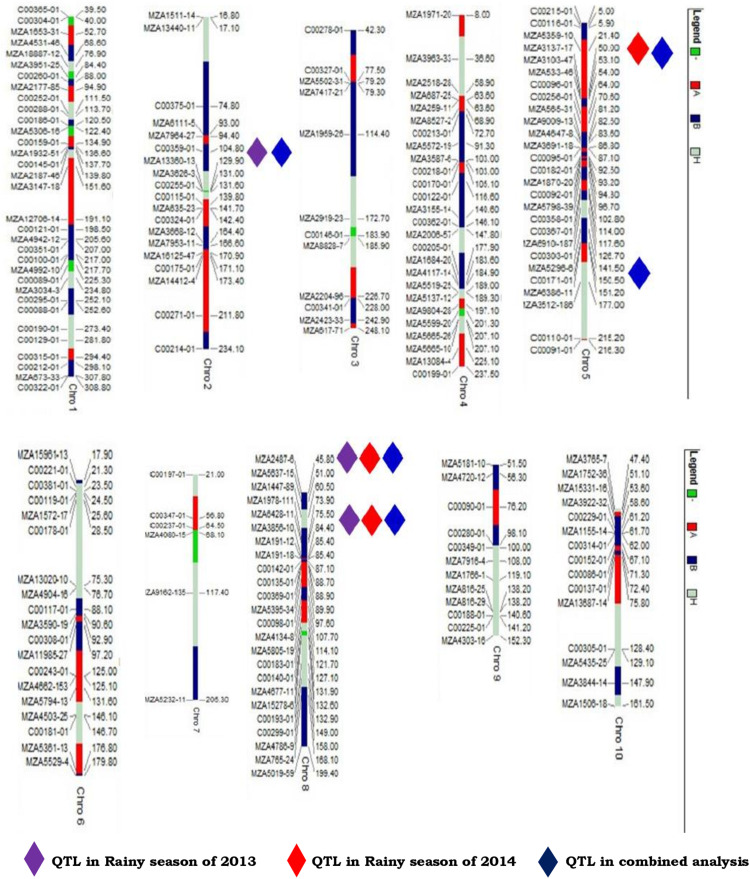
Linkage map and position of the QTL associated with northern corn leaf blight resistance mapped from F2:3 mapping population of the cross CML153 × SKV50.

**FIGURE 3:**
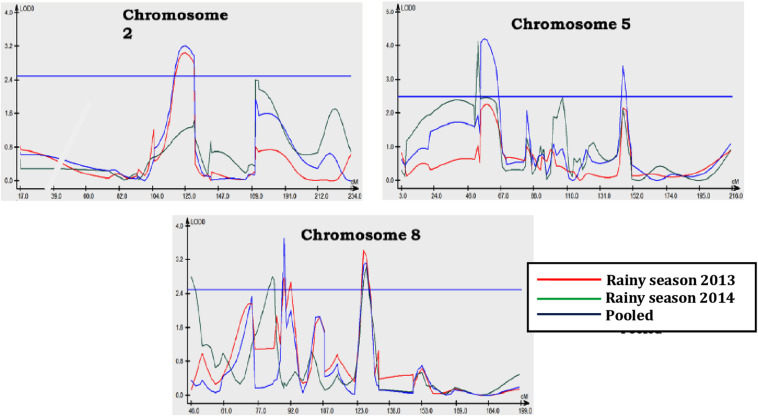
LOD peak for QTL conditioning resistance to northern corn leaf blight on chromosomes 2, 5, and 8 in rainy season *(Kharif)* of 2013, 2014, and pooled analysis over seasons.

Three QTL positions were identified for northern corn leaf blight resistance during the rainy season of 2013 (Table 2 and Figures 2, 3). One QTL was located on chromosome 2 (qNCLB-2) flanked by markers C00359-01–MZA13360-13 which explained 3.07% phenotypic variation with LOD of 3.06. Two QTL were located on chromosome 8 (qNCLB-8-1 and qNCLB-8-2) flanked by markers, viz. MZA2487-6 and MZA6428-11–MZA3856-10, and these two QTL showed phenotypic variation of 2.46 and 22.97% with LOD score of 2.77 and 3.44, respectively. A major northern corn leaf blight QTL was mapped on chromosome 8 (qNCLB-8-2) which explained maximum phenotypic variation of 22.97%. These three identified QTL explained a total of 28.50% phenotypic variation. Additive gene effects of these three QTL ranged from 1.71 to 1.92, and the favorable alleles for these QTL were contributed by the resistant parent SKV50. The QTL located on chromosome 2 (qNCLB-2) and on chromosome 8 (qNCLB-8-2) exhibited overdominance gene action, while another QTL on chromosome 8 (qNCLB-8-1) revealed a dominance type of gene action.

**TABLE 2:** QTL detected for northern corn leaf blight resistance during individual seasons and combined over seasons using 344 F_2__:__3_ families from the cross CML 153 × SKV 50 (threshold LOD score = 2.50).

**Season**	**Chromosome**	**Bin location**	**Flanking markers**		**QTL position (cM)**	**Maximum LOD score**	***R*^2^ (%)**	**Genetic effect**		**Gene action**	**Donor allele**	**QTL name**
			**left**	**Right**				**Additive**	**Dominance**			
Rainy season, 2013	2	2.06	C00359-01	MZA13360-13	108.11	3.06	3.07	1.71	3.02	OD	SKV50	*qNCLB-2*
	8	8.03	MZA2487-6		42.91	2.77	2.46	1.72	2.07	D	SKV50	*qNCLB-8-1*
	8	8.06	MZA6428-11	MZA3856-10	80.01	3.44	22.97	1.92	3.62	OD	SKV50	*qNCLB-8-2*
Rainy season, 2014	5	5.03	MZA5359-10	MZA3137-17	49.21	4.12	1.00	3.48	4.30	OD	SKV50	*qNCLB-5-1*
	8	8.03	MZA2487-6		37.81	2.80	1.90	1.65	0.89	D	SKV50	*qNCLB-8-1*
	8	8.06	MZA6428-11	MZA3856-10	81.31	3.05	9.68	−0.13	3.51	OD	SKV50	*qNCLB-8-2*
Combined	2	2.06	C00359-01	MZA13360-13	108.11	3.22	2.28	1.25	2.33	OD	SKV50	*qNCLB-2*
	5	5.03	MZA3103-47	MZA533-46	53.21	4.21	1.77	0.74	2.41	OD	SKV50	*qNCLB-5-2*
	5	5.04–5.05	MZA5296-6	C00171-01	142.71	3.40	10.24	3.39	−1.20	D	SKV50	*qNCLB-5-3*
	8	8.03	MZA2487-6		43.21	3.71	1.64	2.99	1.07	D	SKV50	*qNCLB-8-1*
	8	8.06	MZA6428-11	MZA3856-10	81.31	3.13	16.34	0.56	2.60	OD	SKV50	*qNCLB-8-2*

*OD, overdominance; D, dominance.*

During the rainy season of 2014, three QTL regions conferring resistance to northern corn leaf blight were mapped onto chromosome 5 and chromosome 8. Among these, a QTL bracketed by markers MZA6428-11–MZA3856-10 on chromosome 8 explained the highest phenotypic variation of 16.8% with LOD score 3.05 (qNCLB-8-2) followed by a QTL on chromosome 8 present adjacent to the marker MZA2487-6 which explained 2.90% of phenotypic variation with LOD score of 2.80 (qNCLB-8-1). Other QTL on chromosome 5 flanked by markers MZA5359-10–MZA3137-17 (qNCLB-5-1) contributed 2.00% to the phenotypic variation with LOD score of 4.12. The range of additive genetic effects for these QTL was from −0.13 to 3.48, and the total phenotypic variation of 21.60% was explained by the QTL identified. Favorable allele for QTL located on chromosome 8 (qNCLB-8-2) was contributed by susceptible parent CML153, whereas for other QTL, it was by the resistant parent SKV50. The QTL (qNCLB-8-1) on chromosome 8 showed dominance gene action and two QTL (qNCLB-8-2 and qNCLB-5-1) located on chromosomes 8 and 5 showed overdominance gene action.

In the combined QTL analysis, five QTL were detected on chromosomes 2, 5, and 8. Among these QTL, three QTL flanked by markers C00359-01–MZA1336013 (qNCLB-2), MZA2487-6 (qNCLB-8-1), and MZA6428-11–MZA3856-10 (qNCLB-8-2) were found to be consistent across two seasons with a LOD score of 3.22, 3.71, and 3.13. Two novel QTL were located on chromosome 5 flanked by markers MZA3103-47–MZA533-46 (qNCLB-5-2) and MZA5296-6–C00171-01 (qNCLB-5-3). The QTL qNCLB-8-2 explained the highest phenotypic variation of 16.34% followed by qNCLB-5-3 (10.24%). A total phenotypic variation explained by these QTL was 32.27% in the pooled analysis. An additive genetic effect of these QTL ranged from 0.74 to 3.39, and the favorable allele for these QTL was contributed by the resistant parent SKV50. Out of five QTL, two QTL (qNCLB-5-3 and qNCLB-8-1) located on chromosomes 5 and 8 exhibited dominance, and the other three QTL (qNCLB-2, qNCLB-5-2, and qNCLB-8-2) on chromosomes 2, 5, and 8 showed overdominance gene action in the combined analysis.

### Co-localization of QTL Conferring Resistance to Multiple Foliar Pathogens

Since QTL analysis for sorghum downy mildew and southern corn rust was performed from one year data, results were not presented. However, the information generated was used for the estimation of pairwise Pearson correlation coefficient between three diseases (northern corn leaf blight, sorghum downy mildew, and southern corn rust) and co-localization of QTL. The highest significant and positive correlation was noticed between northern corn leaf blight and southern corn rust (*r* = 0.122), whereas a positive but non-significant pairwise correlation between sorghum downy mildew and southern corn rust (*r* = 0.061) was observed (Table 3). Between sorghum downy mildew and northern corn leaf blight, a negatively significant correlation was revealed (*r* = −0.120). This clearly indicated the presence of genes carrying resistance to multiple diseases in the F_2__:__3_ population derived from the cross CML153 × SKV50. In the present study, co-localized chromosomal regions harboring QTL for northern corn leaf blight, sorghum downy mildew, and southern corn rust resistance were observed (Table 4). In bin 8.03, QTL conferring resistance to all three foliar diseases, viz. northern corn leaf blight (one QTL), sorghum downy mildew (two QTL), and southern corn rust (two QTL), were co-localized at approximately the same map position (<10 cM difference between the QTL peaks for the three diseases) with the common adjacent marker MZA2487-6. The bin 5.03–5.04 was significantly associated with resistance to northern corn leaf blight and sorghum downy mildew, wherein two QTL for northern corn leaf blight and one QTL for sorghum downy mildew co-localized with a map distance of <3 cM. This co-localized QTL exhibited 10.24 and 5.98% of total phenotypic variation for northern corn leaf blight and sorghum downy mildew, respectively. The QTL for southern corn rust and sorghum downy mildew resistance were co-localized in bin 3.04 at a map distance of 1 cM with a common flanking marker MZA1959-26, while QTL conferring resistance to northern corn leaf blight and sorghum downy mildew were co-localized in chromosome bin 2.06.

**TABLE 3:** Pearson correlation coefficient between mean disease severity for three diseases, viz. northern corn leaf blight (NCLB), sorghum downy mildew (SDM), and southern corn rust (SCR).

**Disease**	**NCLB**	**SDM**	**SCR**
NCLB	1	−0.120*	0.122*
SDM		1	0.061
SCR			1

*Significant at the 5% level of significance.

**TABLE 4:** Co-located QTL conditioning resistance to northern corn leaf blight (NCLB), sorghum downy mildew (SDM), and southern corn rust (SCR).

**Co-localized chromosome bin**	**Disease QTL**	**Flanking marker interval**	**QTL position (cM)**	**Maximum LOD score**	***R*^2^ (%)**	**Genetic effect**		**Gene action**	**Donor Allele**
						**Additive**	**Dominance**		
8.03	NCLB	MZA2487-6	43.21	3.71	1.64	2.99	1.07	D	SKV50
	SDM	MZA2487-6	26.71	5.72	1.20	1.44	9.49	OD	SKV50
	SDM	MZA2487-6	39.61	3.89	1.63	5.23	2.24	PD	SKV50
	SCR	MZA2487-6	22.71	2.92	4.17	1.67	5.23	OD	SKV50
	SCR	MZA2487-6–MZA5637-15	48.21	3.11	4.71	8.19	6.79	D	SKV50
5.03/5.04	NCLB	MZA3103-47–MZA533-46	53.21	4.21	1.77	0.74	2.41	OD	SKV50
	NCLB	MZA5296-6–C00171-01	142.71	3.40	10.24	3.39	−1.20	D	SKV50
	SDM	C00303-01–MZA5296-6	138.71	3.22	5.98	3.44	−2.23	PD	SKV50
3.04	SDM	MZA7417-21–MZA1959-26	100.11	2.56	8.28	8.13	−12.24	OD	SKV50
	SCR	MZA7417-21–MZA1959-26	102.11	9.46	1.01	−16.28	15.09	D	CML153
2.06	NCLB	C00359-01–MZA13360-13	108.11	3.22	2.28	1.25	2.33	OD	SKV50
	SDM	C00324-01–MZA3668-12	147.61	2.57	20.42	3.11	1.09	PD	SKV50
	SDM	C00324-01–MZA3668-12	154.31	2.63	15.43	−2.88	1.35	PD	CML153

*PD, partial dominance.*

### Development of Inbred Lines With Resistance to Three Diseases With Good General Combining Ability

We developed 125 lines and crossed with the broad genetic base tester CM500 for testing their combining ability. Only 77 lines produced successful test cross progenies. The evaluation of these test cross progenies resulted in the identification of 39 lines with high general combining ability for grain yield with 17 lines showing significant standard heterosis. These 77 lines exhibited resistance/moderate resistance to NCLB, SDM, and Polysora rust diseases (Supplementary Table 1).

## Discussion

Highly diverse parents for multiple pathogen infection were used in the development of F_2__:__3_ progenies which resulted in highly significant differences among the progenies for disease reaction against northern corn leaf blight pathogen. The significant difference among means of F_3_ families for northern corn leaf blight indicated the presence of genotypic variability within the population. Assuming a random effects model, Bartlett’s test proved the homogeneity of error mean sum of squares for northern corn leaf blight data between 2013 and 2014. Therefore, data from these two seasons were pooled. In the pooled analysis, genotype × season interaction was significant, demonstrating the influence of season on northern corn leaf blight incidence (Chen et al., 2016; Shridhar Hegde et al., 2018). In this study, disease pressure was high in 2013 compared with 2014. A significant seasonal effect was observed which indicated that disease development was highly influenced by weather conditions like rainfall, temperature, and relative humidity. Weather data recorded at the V.C. Farm, Mandya indicated comparatively more number of rainy days coupled with high humidity during 2013 resulting in a higher range of disease expression.

Frequency distribution of 344 F_3_ progenies from the cross CML153 × SKV50 revealed non-normal distribution for the incidence of NCLB. Positively skewed distribution was observed for northern corn leaf blight in each season data and pooled data. Skewed distribution was observed toward the resistant parent SKV50 indicating the dominance of resistance (Schechert et al., 1999; Welz et al., 1999; Brown et al., 2001; Asea et al., 2009). However, the distribution was made near normal through arcsine transformation of the percent disease incidence data. Approximately or near normal distribution in phenotypic data was obtained earlier on F_2__:__3_ populations (Holland et al., 1998; Brunelli et al., 2002; Shridhar Hegde et al., 2018). The resistance to NCLB disease appeared to be controlled by a larger number of genes having decreasing effects with the involvement of dominance-based complementary interaction as evidenced by platykurtic and positively skewed distribution (Pooni et al., 1977; Choo and Reinbergs, 1982). High heritability with high genetic advance over mean indicated the reliability of the estimates of variation between F_3_ families, and a reasonable progress in selection is possible for disease resistance in this population. Similar results were reported for northern corn leaf blight by Freymark et al. (1993, 1994), Schechert et al. (1999), Hakiza et al. (2004), Balint-Kurti et al. (2010), and Zwonitzer et al. (2010).

In the present investigation, 194 SNP markers showed the expected Mendelian segregation ratios and were used to construct relatively high density linkage map consisting of 10 linkage groups. The total map distance covered about 2,143.02 cM with an average interval length of 10.77 cM. The length of a linkage map is influenced by a number of factors, such as the number of markers, size of the mapping population, and genotyping accuracy. The average distance between markers in our linkage map was 10.77 cM, which was longer than the other saturated linkage maps with SNP markers (729.28–2,236.66 cM in length with an interval of 0.66–10 cM) which were successfully utilized for the identification of QTL for various traits in maize (Zou et al., 2012; Chen et al., 2016; Zhou et al., 2016; Su et al., 2017; Xia et al., 2020). However, Doerge (2002) was of the opinion that QTL mapping of important traits can be practiced with marker density up to 20 cM. The near normal distribution pattern of F_3_ progenies suggested the reliability of the F_2__:__3_ mapping population for the identification of QTL for resistance to northern corn leaf blight.

### Mapping of QTL Conferring Resistance to Northern Corn Leaf Blight

The identification of QTL with resistance to diseases could serve as a novel strategy to develop disease-resistant lines. The SNP-based linkage maps have been used to precisely map disease resistance through genetic linkage and extensively helped researchers in understanding the function of the chromosomal region or locus at gene levels (St Clair, 2010). In this study, genomic locations of QTL for northern corn leaf blight were identified on chromosomes 2, 5, and 8. The QTL on chromosomes 5 and 8 were major with a high percentage of the phenotypic variance for resistance. The QTL detected on chromosome 8 in bin 8.06 explained 16.34% of the phenotypic variance followed by the QTL on chromosome 5 which explained 10.24% variation in the combined analysis. When considered together, the total variance explained by these QTL was 26.58%. The UMC reference map of maize (Davis et al., 1999) was used to compare QTL positions (Lin et al., 1995; Tuberosa et al., 2002). The major QTL on chromosome bins 5.04–5.05 and 8.06 were also reported by Freymark et al. (1993, 1994) in bins 5.03–5.05 and 8.03–8.06, Dingerdissen et al. (1996) in bins 5.03 and 8.06, Schechert et al. (1999) in bins 5.03–5.05 and 8.06, Welz et al. (1999) in bins 5.03–5.04 and 8.02–8.06, Welz and Geiger (2000) in bins 5.04 and 8.06, Brown et al. (2001) in bin 8.06, Asea et al. (2009) in bins 5.04 and 8.06, Chung et al. (2011) in bins 5.03 and 8.06, and Chen et al. (2016) in bin 5.04. This supports the suggestion from Chung et al. (2010), who fine-mapped northern corn leaf blight QTL on chromosome bin 8.06, that a major QTL region on chromosome 8 affects the response to northern corn leaf blight. Similarly, an important role of QTL on chromosome 5 at bin 4 was deciphered as an additional QTL to that already known in bins 5.03, 5.04, and 5.05 by Welz et al. (1999), Asea et al. (2009), and Chung et al. (2011). Another QTL detected in chromosome bin 2.06 was also reported earlier by Schechert et al. (1999), Welz et al. (1999), Brown et al. (2001), Ping et al. (2007), Balint-Kurti et al. (2010), Zwonitzer et al. (2010); and Shridhar Hegde et al. (2018). The major QTL identified in our study at bin location 8.06 exhibited overdominance (OD) gene action. It could be confirmed that northern corn leaf blight resistance alleles in SKV50 are a good source for marker-assisted selection supporting maize breeding programs. Similar findings were observed by Welz et al. (1999), Welz and Geiger (2000), Brown et al. (2001), Chung et al. (2010), Zwonitzer et al. (2010), and Chung et al. (2011).

One of the major goals of QTL mapping is to identify and use QTL with little QTL × environment interaction (Stuber et al., 1987). This was substantiated in the present study as QTL in bins 8.03 and 8.06 were detected in both seasons and in the pooled analysis. Earlier workers also reported the identification of QTL in all environments with difference in the level of significance and magnitude of genetic effects (Brown et al., 2001; Asea et al., 2009; Balint-Kurti et al., 2010; Chen et al., 2016; Shridhar Hegde et al., 2018). Conclusively, the markers associated with QTL in bins 2.06 (C00359-01 and MZA13360-13), 5.03 (MZA3103-47 and MZA533-46), 5.04 (MZA5296-6 and C00171-01), and 8.06 (MZA6428-11 and MZA3856-10) are the favorites to be used to transfer resistance alleles to susceptible lines (Xia et al., 2020). The QTL localized in chromosome bin 5.04 was previously detected in the NAM population (Poland et al., 2011) and is known to contain a candidate gene, GRMZM2G024612 (Xia et al., 2020). The genomic region in chromosome 8 is important as two resistant genes *Ht2* and *Htn1* were identified previously (Zaitlin et al., 1992; Simcox and Bennetzen, 1993; Hurni et al., 2015; Kaefer et al., 2017). Wisser et al. (2006) reported *Histatin-1* (*Htn1*) as a maize disease resistance gene against NCLB and is known to encode a wall-associated receptor-like kinase that acts as an important component of the plant innate immune system by perceiving pathogen or host-derived elicitors (Hurni et al., 2015). The gene *Ht2* has been further delimited to a region of up to 0.46 Mb *via* precise mapping, and other candidate genes were predicted and annotated according to the reference genome sequences of B73 (Chung et al., 2010). Except for chromosomes 5 and 8, NCLB resistance-related QTL seems to be relatively rare at other chromosomes (Xia et al., 2020).

Clearly, there is a lack of commonality between the QTL identified for northern corn leaf blight in different populations. This could be because of multiple reasons such as the use of different types and sizes of mapping populations, involvement of different sets of QTL in different crosses, and epistatic interaction between QTL. Furthermore, as noted in previous studies, it is possible that QTL may not be detected in certain segregating populations if alternate alleles of the QTL are not contributed by both parents (Beavis and Keim, 1996; Kearsey and Pooni, 1996; Bohn et al., 1997). In a previous study, a comparison of QTL for disease resistance in multiple segregating populations revealed only few common QTL (Beavis et al., 1991). This is primarily due to environmental conditions existing in a particular region which might affect the QTL expression. Nonetheless, the detection of different QTL present in diverse resistant genotypes could aid in the pyramiding of multiple QTL in cultivars.

### Co-localization of QTL Conferring Resistance to Three Foliar Diseases

Mapping resistance loci for multiple pathogens provides an opportunity for co-localizing resistance loci. Such resistance gene combinations are expected to provide more durable protection (Simmonds, 1985) against a variable number of pathogens. In the present study, QTL for northern corn leaf blight, sorghum downy mildew, and southern corn rust resistance were co-localized based on the bin locations and chromosomal regions where QTL were detected. Associations between resistance to northern corn leaf blight, sorghum downy mildew, and southern corn rust were significant in the F_2__:__3_ population. These associations were detected most likely because of alleles showing a high level of resistance to one disease and a lower level to another which may not be detected by QTL analysis. Alternatively, it could be because of alleles that confer varied levels of resistance to multiple diseases that are undetectable by QTL analysis. A total of 13 QTL associated with resistance to two or more diseases were detected. These QTL regions may carry a single gene showing multiple disease resistance or due to two closely linked genes causing resistance to different diseases in the F_2__:__3_ population. The inbred SKV50 with resistance to foliar diseases, viz. northern corn leaf blight, sorghum downy mildew, and southern corn rust, could be used for pyramiding resistance QTL. The study also indicated the potential of using several target QTL present in chromosome bin 8.03 for resistance to northern corn leaf blight, sorghum downy mildew, and southern corn rust: bins 5.03–5.04 and 2.06 for resistance to northern corn leaf blight and sorghum downy mildew and bin 3.04 for resistance to sorghum downy mildew and southern corn rust for marker-assisted selection to pyramid quantitative resistance to multiple foliar pathogens as described by Pratt and Gordon (2006).

The present study resulted in the identification of markers linked to resistance to northern corn leaf blight, and some common QTL for resistance to three important foliar diseases were identified. It was also possible to validate the major QTL linked to NCLB, SDM, and SCR and to develop 39 inbred lines with high general combining ability which could be used in hybrid development programs.

## Data Availability Statement

The original contributions presented in the study are included in the article/Supplementary Material, further inquiries can be directed to the corresponding author/s.

## Author Contributions

HL and HR designed and carried out the experiments and contributed to manuscript preparation. AP helped in genotyping of the mapping populations and editing of the manuscript. All authors contributed to the article and approved the submitted version.

## Conflict of Interest

AP is employed by company Corteva Agriscience Pvt. Ltd. The remaining authors declare that the research was conducted in the absence of any commercial or financial relationships that could be construed as a potential conflict of interest.

## Footnotes

1 www.panzea.com

2 http://www.illumina.com/

3 www.maizegdb.org/bin-viewer

4 www.panzea.org

5 www.maizeGDB.org
